# Supplementary material for: Pharmacological induction of selective endoplasmic reticulum retention as a strategy for cancer therapy
Source: Nat Commun. 2020 Mar 11;11:1304. doi: 10.1038/s41467-020-15067-5 (PMC7066181; doi:10.1038/s41467-020-15067-5)
Supplement: Supplementary file 1 — Supplementary Information [file 41467_2020_15067_MOESM1_ESM.pdf]

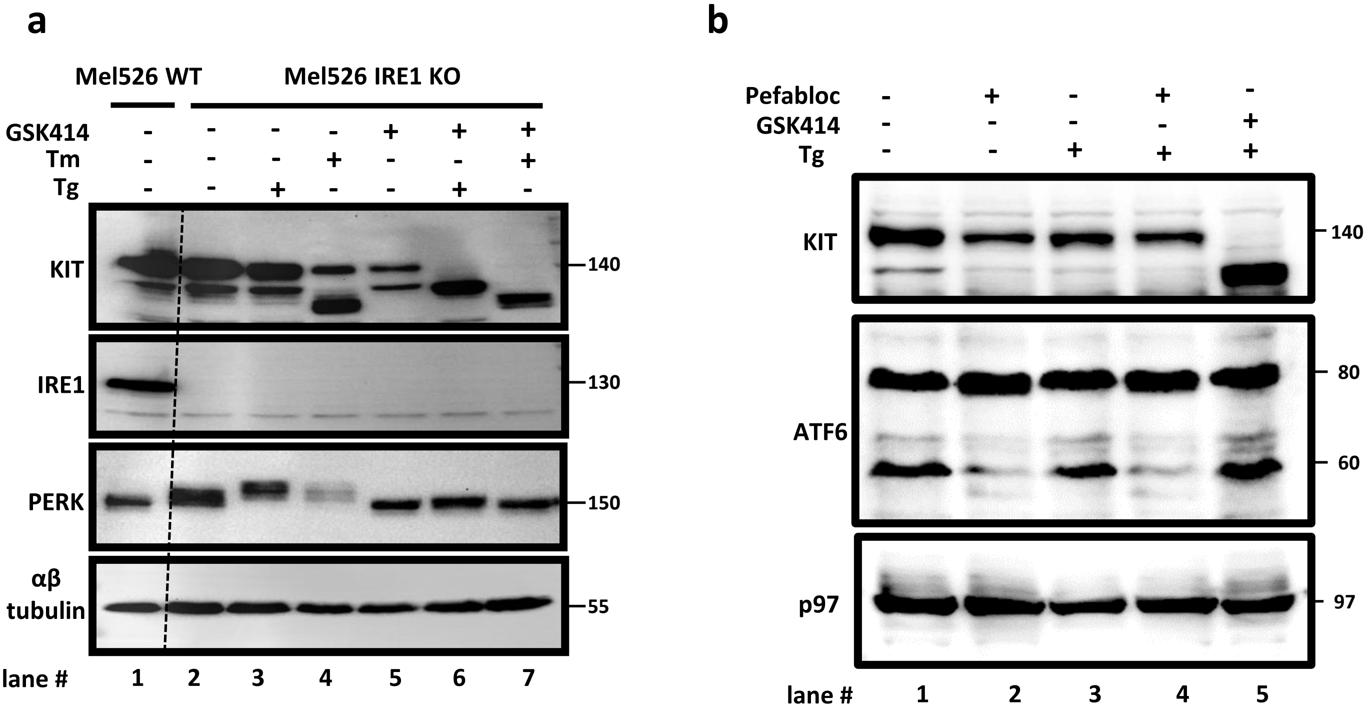

Supplemental figure 1: Inactivation of IRE1 or ATF6 does not confer the retention of KIT in the ER upon PERK inhibition. a Immunoblotting of Mel526 WT and IRE1 KO cells followed treatment with DMSO, Tg, or Tm alone or in combination with GSK414 (0.5 μM) for 16 hours; **b** Immunoblotting of Mel526 WT cells treated with DMSO, Tg, GSK414, and Pefabloc (300 μM) alone or in combination for 16 hours. Shown are typical experiments of two independent repetitions.

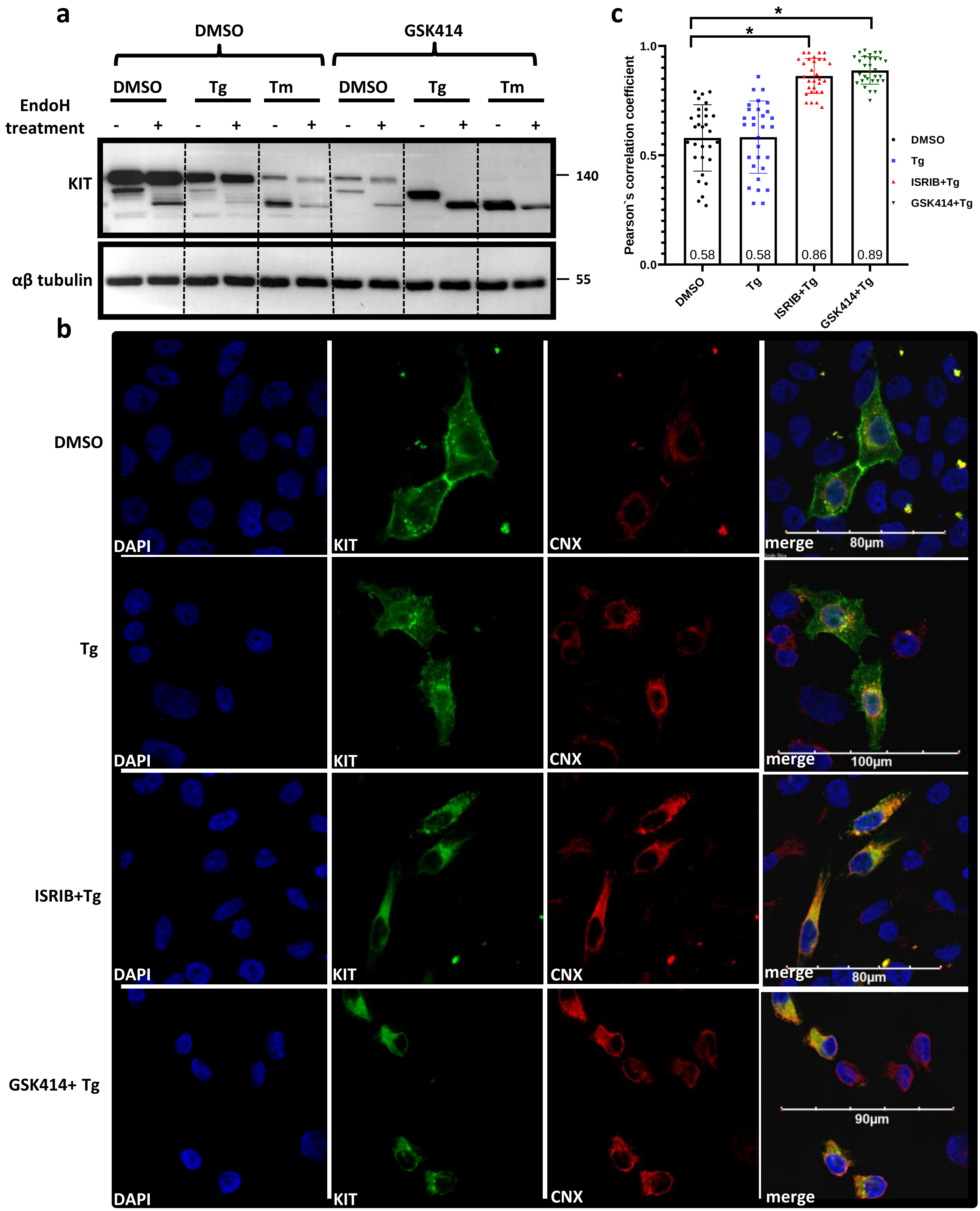

Supplemental figure 2: KIT is arrested in the ER, when ER stress is applied and the PERK/ISR pathway is inhibited a Mel526 cells were treated either with DMSO, GSK414, Tg, or Tm alone or with combinations for 16 hours. The extracted lysates were then incubated with EndoH followed by immunoblotting for KIT and αβ tubulin as a loading control; Shown one of two repetitions with a similar outcome; **b** Immunofluorescence microscopy for the nucleus DAPI (blue), KIT (anti-FLAG, green), and calnexin (CNX, red) of KIT KO Mel526 cells transfected with KIT-3xFLAG expressing vector following DMSO, Tg, Tg/ISRIB, or Tg/GSK414 treatments for 12 hours. **c** Evaluation of colocalization by Pearson's coefficient and two-tailed Student T test for statistical significance ± SD. **p*<0.01. N=20.

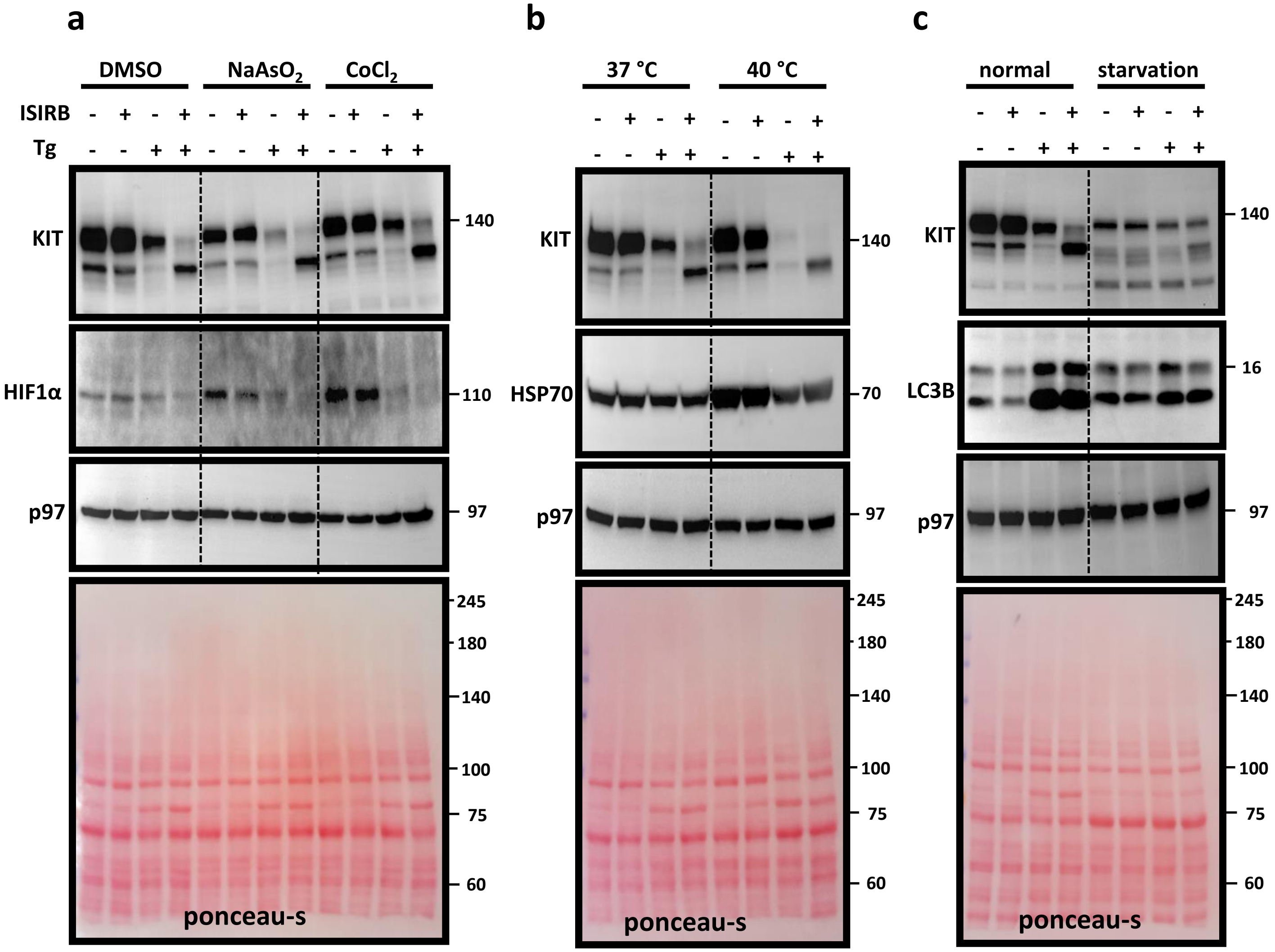

Supplemental figure 3: ISR inhibition leads to ER retention of KIT only when it is accompanied by ER stress. **a** Ponceau-s staining and immunoblotting for KIT, HIF1α, and p97 as a loading control of Mel526 treated with DMSO, Tg, ISRIB, or Tg/ISRIB incubated either under DMSO, sodium arsenite (4 μM), or cobalt chloride (200 μM) for 12 hours; **b** Immunoblotting for KIT, HSP70, and p97 for treated Mel526 incubated either in 37 °C or in 40 °C for 12 hours; **c** Immunoblotting for KIT, LC3B, and p97 of treated Mel526 cells that were incubated either in complete medium or serum-free medium for 12 hours. Experiments were done once with the internal controls of stress induction.

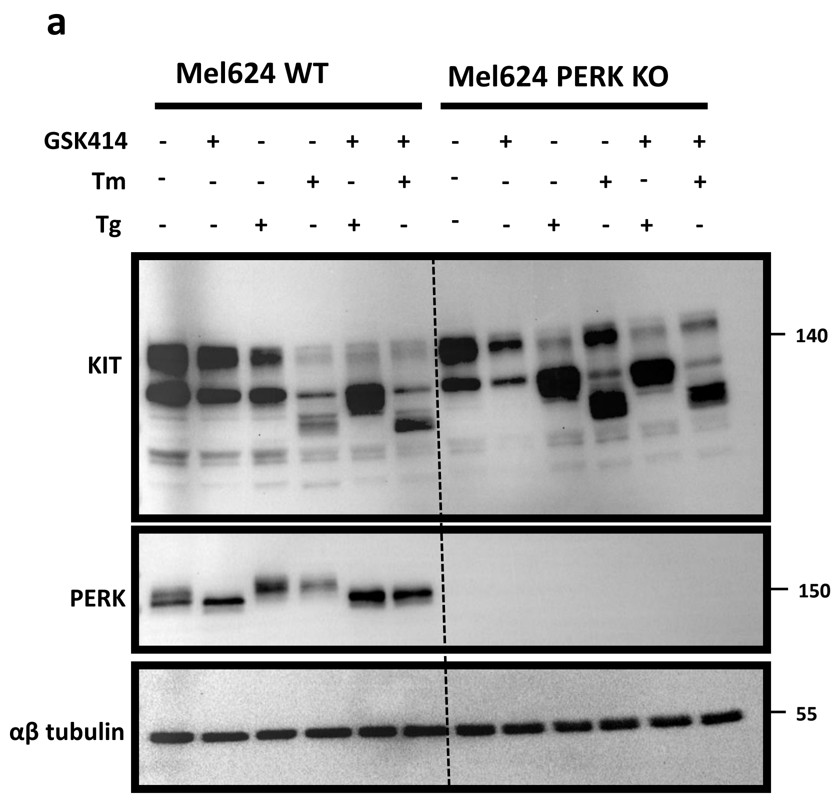

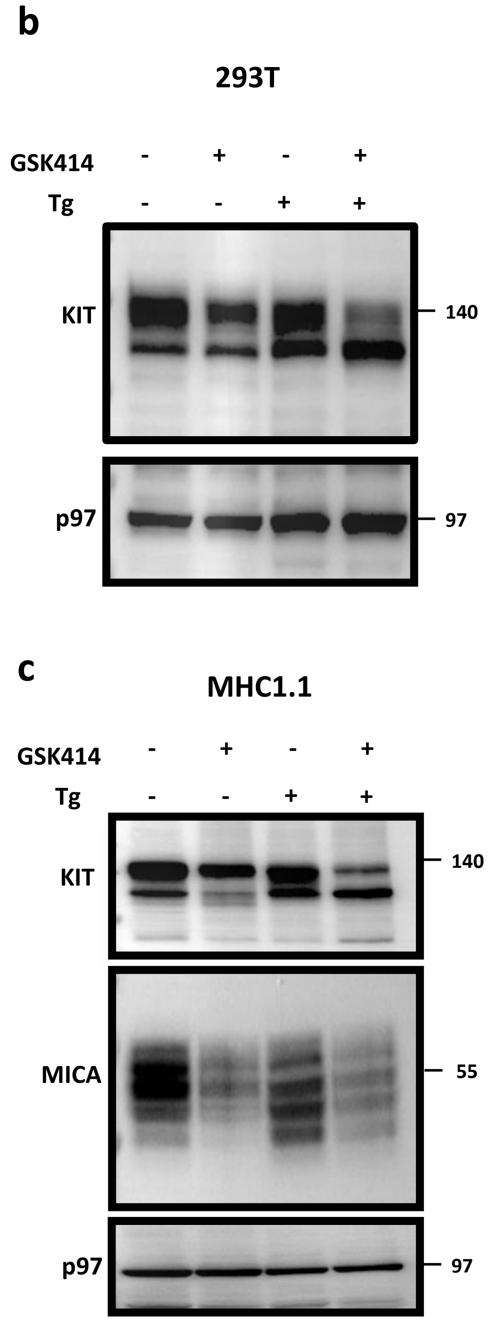

Supplemental figure 4: The ER retention of KIT by Tg/GSK414 treatment is not cell type specific. a Mel624 WT or PERK KO cells were transiently transfected with hKIT expression vector. Once recovered, cells were treated with for GSK414, Tg, and Tm alone or in combinations for 12 hours followed by immunoblotting for KIT, PERK, and αβ tubulin; **b+c** KIT, MICA, and p97 immunoblotting of 293T and HMC1.1 cells following the pharmacological treatments for 12 hours. Shown is one of two experiments with a similar outcome.

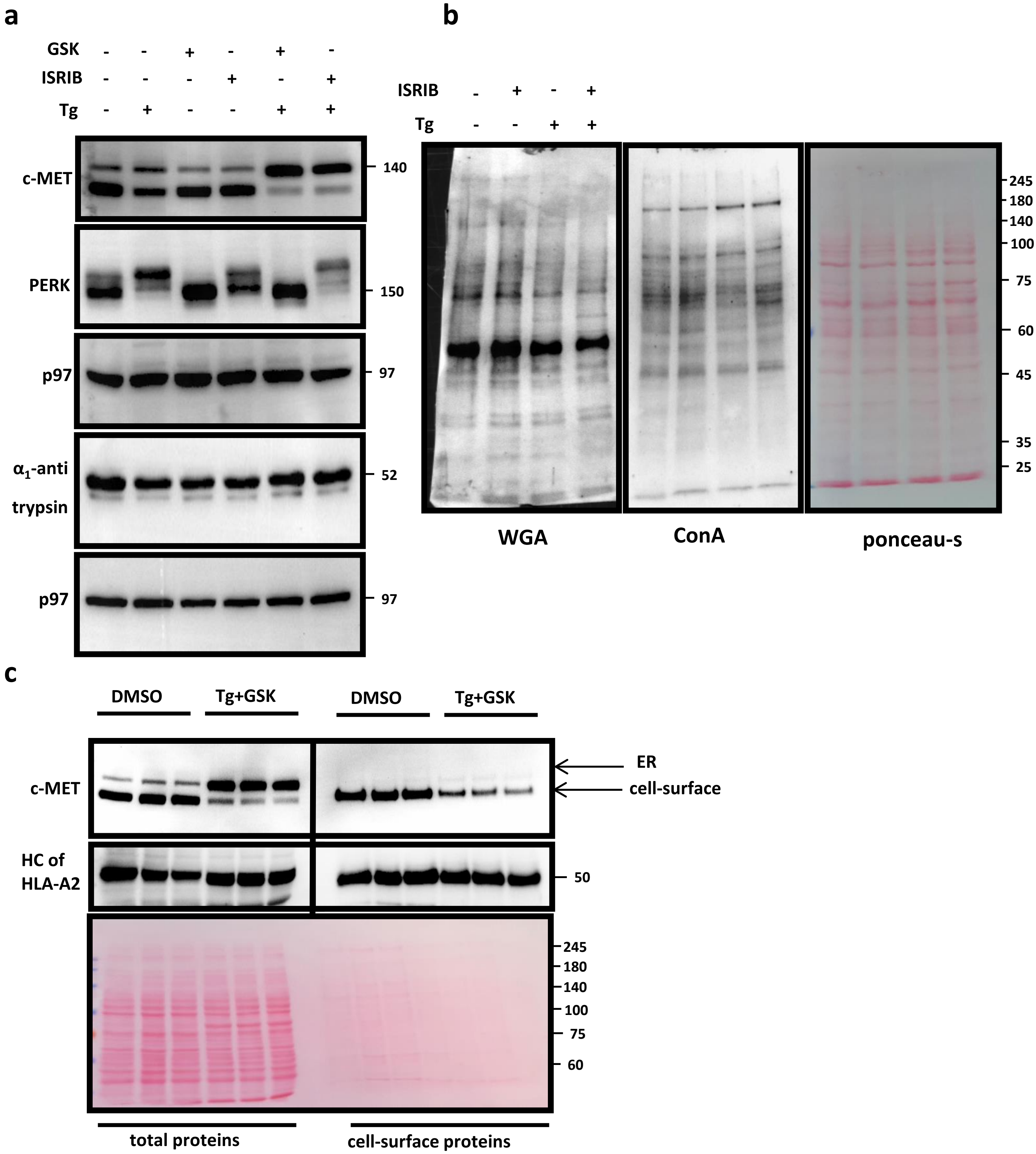

Supplemental figure 5: ER retention by PERK/ISR inhibition accompanied with ER stress is selective. a Immunoblotting for c-MET, PERK, α_1-antitrypsin, and p97 as loading control of hepG2 WT cells following GSK414, ISRIB, and Tg treatments for 16 hours. Shown is one of three independent repetitions; **b** Ponceau-s staining and immunoblotting against total WGA and ConA of Mel526 cells following different treatments with Tg and ISRIB for 12 hours. Shown is one of three independent repetitions. **c** Total lysates and cell-surface glycoproteins fractions immunoblotting against c-MET and HC of HLA-A2 of HepG2 WT following treatment either with DMSO or with Tg/GSK414 for 16 hours. Data are shown as three biological triplicate performed in parallel.

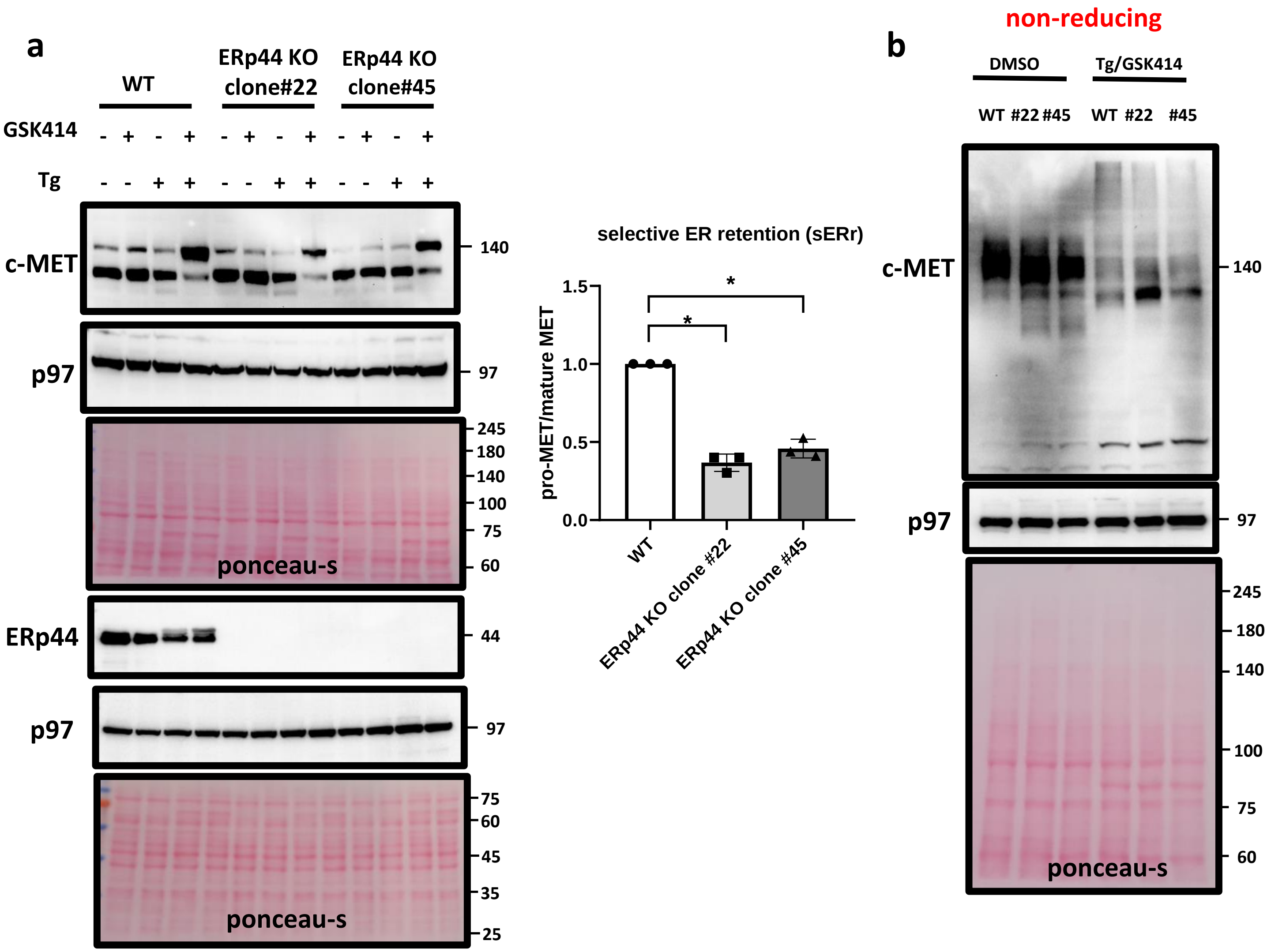

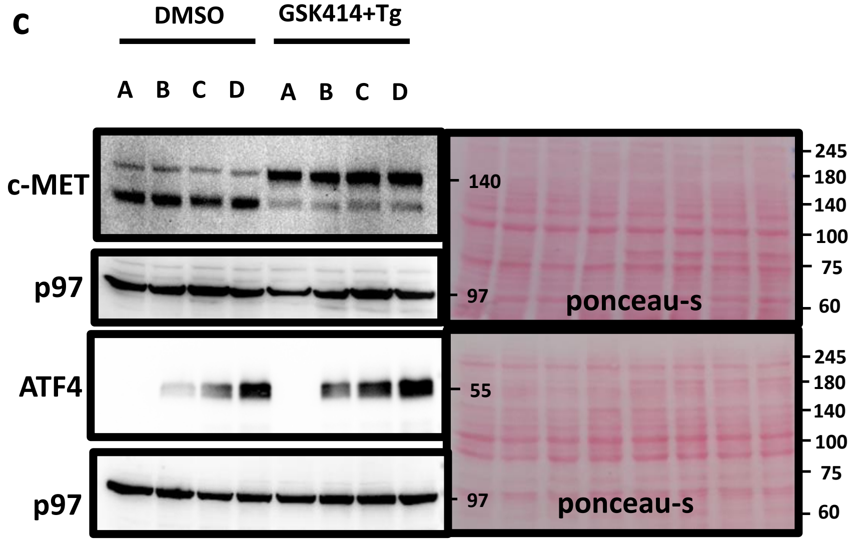

Sample	hATF4 (µg)	Empty pcDNA3.1(+) (µg)
A	0	9
B	1.5	7.5
C	3	6
D	9	0

Supplemental figure 6: ERp44 is involved in sERr efficiency. ATF4 is probably not. a Immunoblotting for c-MET, ERp44, and p97 of HepG2 WT or ERp44 KO lysates followed treatment with Tg and GSK414 for 16 hours. Quantification of c-MET retention efficiency is estimated as the ratio between the pro-MET to the mature form (middle). Shown is average ± SD of N=3. **b** Reducing and non-reducing immunoblotting for c-MET and p97 of HepG2 WT or ERp44 KO cells treated either with DMSO or with Tg+GSK414 for 16 hours. Shown is one of two experiments with a similar outcome. **c** Immunoblotting for c-MET, ATF4, and p97 for HepG2 WT cells that were transfected with increasing amounts of hATF4 expression vector and empty pcDNA3.1 (+) (see table). Once recovered, cells were equally divided and treated either with DMSO or with Tg/GSK414 for 16 hours. Experiment was done once in a dose escalation manner.

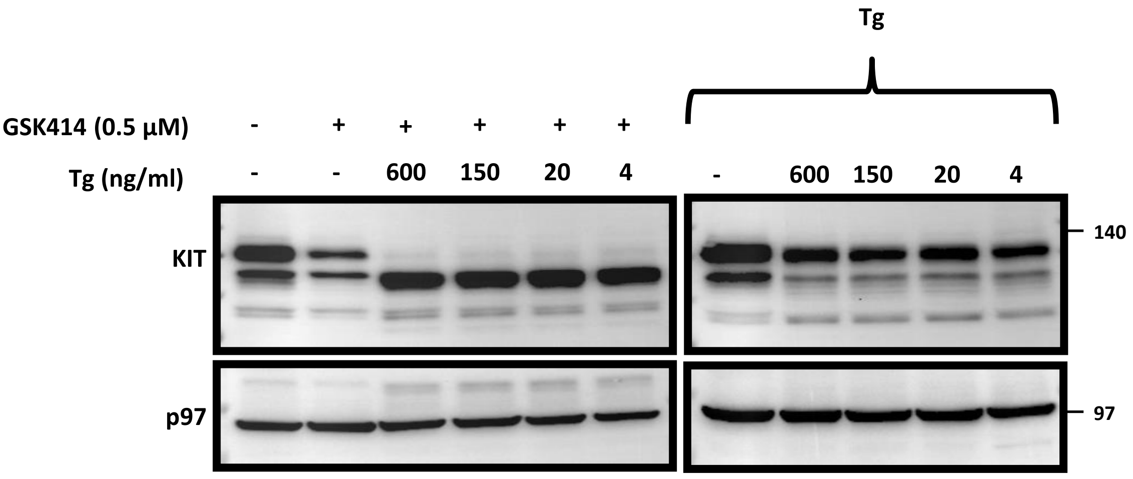

Supplemental figure 7: Mild ER stress is sufficient for SERR. Immunoblotting for KIT and p97 for Mel526 WT lysates following a decreasing concentration of Tg alone (right) or in combination with the 0.5 µM GSK414 for 16 hours. Experiment was done once in a concentration dependent manner.

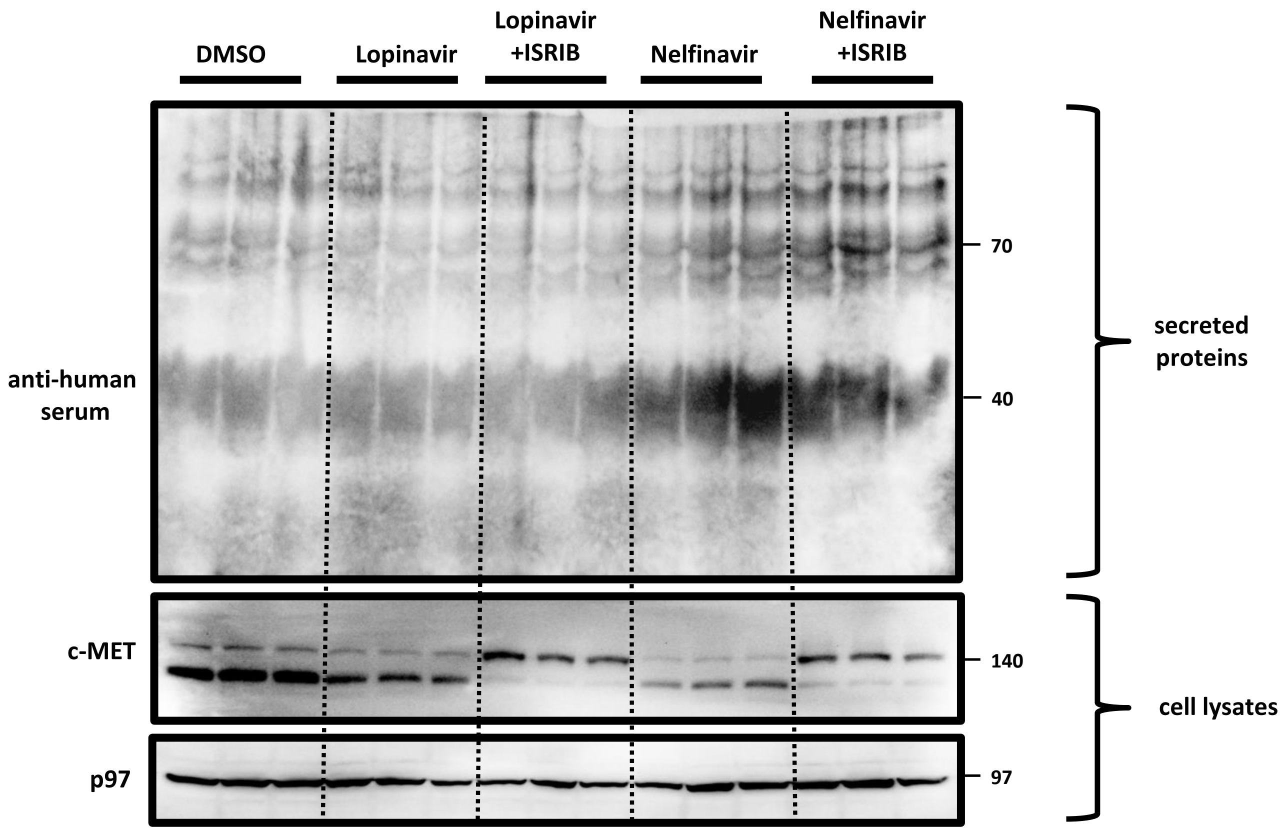

Supplemental figure 8: Pharmacologically induced sERr does not impair cellular secretion in HepG2 cells. HepG2 cells were treated either with Lopinavir (14µM**)** or with Nelfinavir (10µM**)** alone or in combination with ISRIB (0.2µM) in serum-free DMEM for 16 hours. Supernatants were collected and total proteins were isolated by TCA-DOC protein precipitation followed by immunoblotting against human serum. To ensure that drug treatments have induced sERr, lysates were immunoblotting for c-MET and p97 as a loading control. Data are shown as three independent biological experiments.

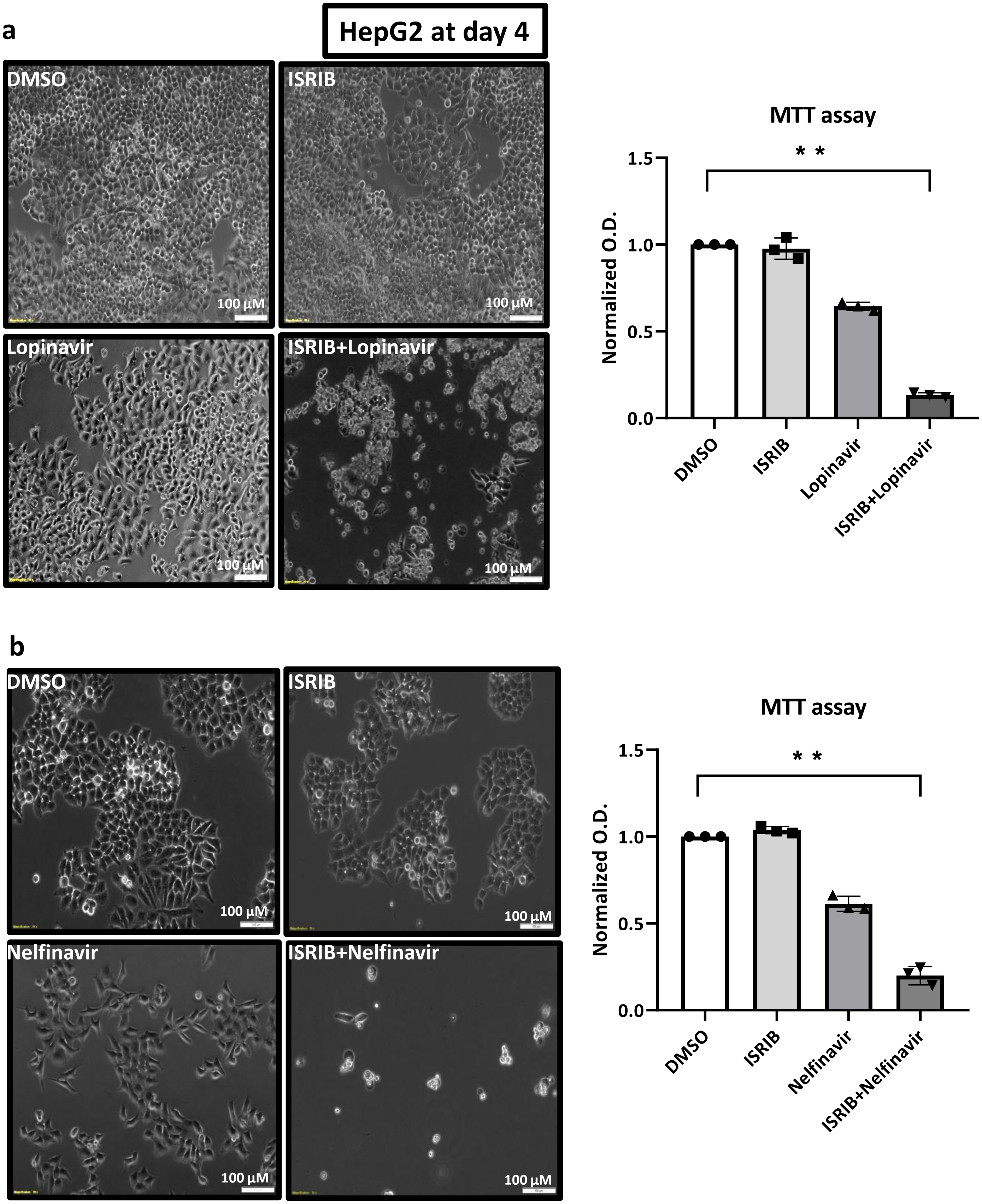

Supplemental figure 9: Dual treatment with lopinavir or nelfinavir in combination with ISRIB compromises the viability of HepG2 cells. a Microscopy images (left) and MTT viability assay (right) of HepG2 cells four days following the treatment with DMSO, ISRIB (0.2 µM) , lopinavir (14 µM), or ISRIB+Lopinavir; **b** Same as in A with nelfinavir (14 µM). MTT averages ± SD of three independent experiments, each performed in triplicates. **p-value <0.01, calculated using ANOVA (two-tailed).

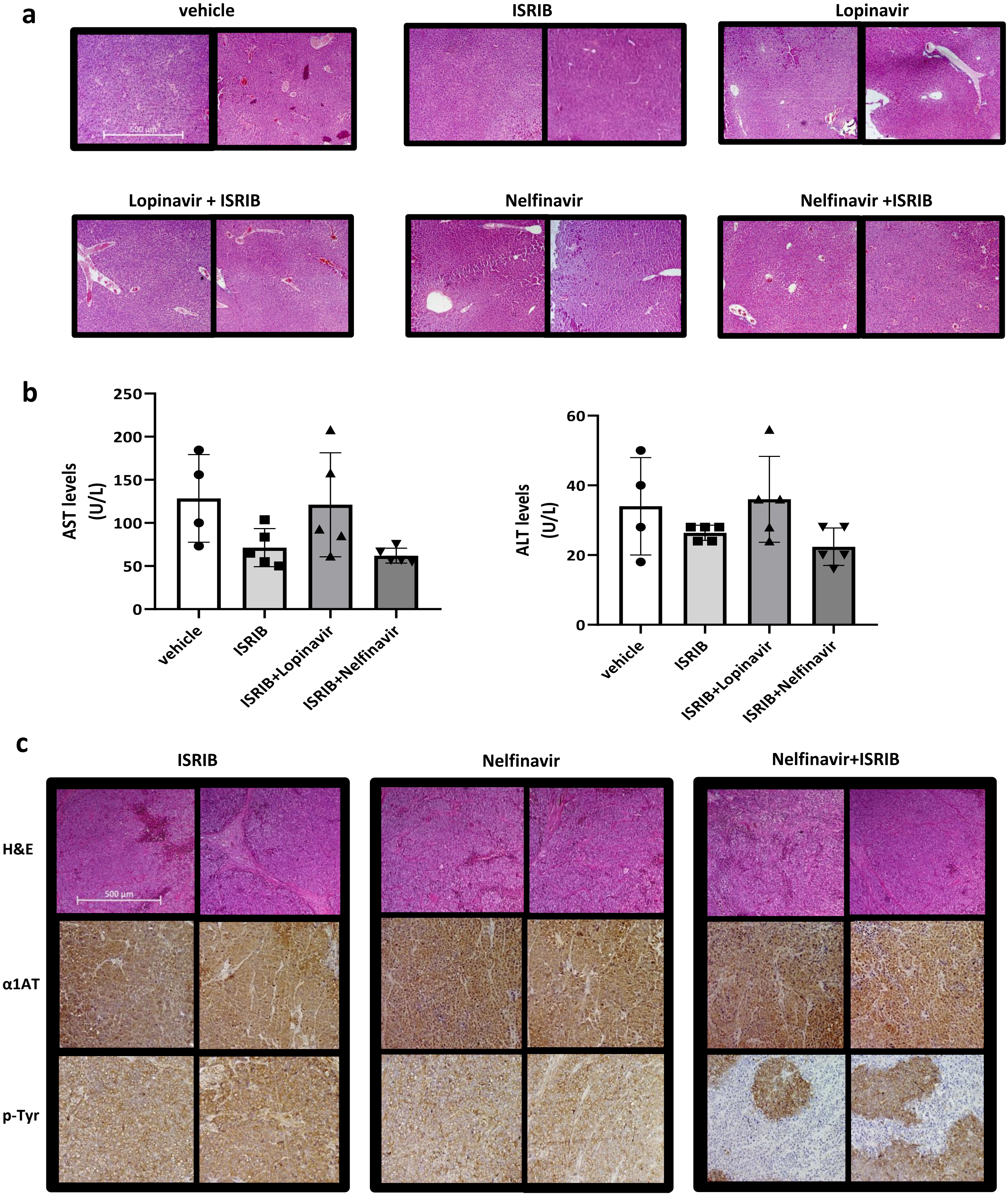

Supplemental figure 10: ISRIB/Nelfinavir treatment significantly reduces tumor P-Tyr levels, with no marked liver toxicity in NOD/SCID mice. a livers H&E staining and **b** AST /ALT levels of tumor bearing mice following drugs injection once a day for 14 days. Shown are averages ± SD of different mice sera, calculated using ANOVA (two-tailed) with p<0.05 for significance. **c** Tumor H&E and immunohistochemistry (IHC) staining against α1AT and P-Tyr following drugs administration twice a day for three consecutive days.
