# Supplementary material for: Pharmacological induction of selective endoplasmic reticulum retention as a strategy for cancer therapy
Source: Nat Commun. 2020 Mar 11;11:1304. doi: 10.1038/s41467-020-15067-5 (PMC7066181; doi:10.1038/s41467-020-15067-5)
Supplement: Supplementary file 3 — Description of Additional Supplementary Information [file 41467_2020_15067_MOESM3_ESM.docx]

**Description of Additional Supplementary Files**

**File Name:** Supplementary Data 1
**Description:** List of all identified proteins.

**File Name:** Supplementary Data 2
**Description:** List of deferentially expressed proteins.
