# Supplementary material for: Pharmacological induction of selective endoplasmic reticulum retention as a strategy for cancer therapy
Source: Nat Commun. 2020 Mar 11;11:1304. doi: 10.1038/s41467-020-15067-5 (PMC7066181; doi:10.1038/s41467-020-15067-5)
Supplement: Supplementary file 6 — Reporting Summary [file 41467_2020_15067_MOESM6_ESM.pdf]

nature research

Corresponding author(s): Boaz Tirosh
DBPR Manuscript # NCOMMS-19-27089A

Last updated by author(s): Dec 15, 2019

Reporting Summary

Nature Research wishes to improve the reproducibility of the work that we publish. This form provides structure for consistency and transparency in reporting. For further information on Nature Research policies, see Authors & Referees and the Editorial Policy Checklist .

Statistics

For all statistical analyses, confirm that the following items are present in the figure legend, table legend, main text, or Methods section.

n/a	Confirmed	
☐	☒	The exact sample size (*n*) for each experimental group/condition, given as a discrete number and unit of measurement
☐	☒	A statement on whether measurements were taken from distinct samples or whether the same sample was measured repeatedly
☐	☒	The statistical test(s) used AND whether they are one- or two-sided<br>*Only common tests should be described solely by name; describe more complex techniques in the Methods section.*
☒	☐	A description of all covariates tested
☒	☐	A description of any assumptions or corrections, such as tests of normality and adjustment for multiple comparisons
☒	☐	A full description of the statistical parameters including central tendency (e.g. means) or other basic estimates (e.g. regression coefficient) AND variation (e.g. standard deviation) or associated estimates of uncertainty (e.g. confidence intervals)
☐	☒	For null hypothesis testing, the test statistic (e.g. *F*, *t*, *r*) with confidence intervals, effect sizes, degrees of freedom and *P* value noted<br>*Give P values as exact values whenever suitable.*
☒	☐	For Bayesian analysis, information on the choice of priors and Markov chain Monte Carlo settings
☒	☐	For hierarchical and complex designs, identification of the appropriate level for tests and full reporting of outcomes
☐	☒	Estimates of effect sizes (e.g. Cohen's *d*, Pearson's *r*), indicating how they were calculated

Our web collection on statistics for biologists contains articles on many of the points above.

Software and code

Policy information about availability of computer code

Data collection	Western blot imaging was performed by BIO-RAD Chemi DOC XRS.<br>Flow cytometry was performed by BECKMAN COULTER CytoFLEX.<br>Confocal imaging was performed by OLYMPUS FLUOVIEW FV10i.<br>Mass spectrometry was done using Q Exactive plus mass spectrometer coupled with Ultimate 3000 LC system<br>Mass spectrometry data was acquired using the Thermo Fisher Scientific software Exactive Series2.9 / Xcalibur 4.1
Data analysis	For western blot : BIO-RAD Image Lab 5.1<br>Flow cytometry: BECKMAN COULTER CytExpert 2.3<br>Confocal microscopy : OLYMPUS FLUOVIEW Ver.2.0c Viewer.<br>Prism8 - GraphPad- for statistical analysis and graphs presentation.<br>ImageJ - For WB quantification and for calculation of Pearson's correlation coefficient for immunofluorescence colocalization analysis.<br>Proteomics: MaxQuant v1.6.3.3 with the human Uniprot database version 01/2019 only canonical and reviewed entries and Perseus software. DAVID webserver was used for human database as background.

For manuscripts utilizing custom algorithms or software that are central to the research but not yet described in published literature, software must be made available to editors/reviewers. We strongly encourage code deposition in a community repository (e.g. GitHub). See the Nature Research guidelines for submitting code & software for further information.

Data

Policy information about availability of data

All manuscripts must include a data availability statement. This statement should provide the following information, where applicable:

- Accession codes, unique identifiers, or web links for publicly available datasets
- A list of figures that have associated raw data
- A description of any restrictions on data availability

The mass spectrometry proteomics data have been deposited in the ProteomeXchange Consortium (http://proteomecentral.proteomexchange.org) via the PRIDE partner repository, with the data set identifier PXD014709.
DAVID webserver was used for human database as background.

Field-specific reporting

Please select the one below that is the best fit for your research. If you are not sure, read the appropriate sections before making your selection.

☒ Life sciences ☐ Behavioural & social sciences ☐ Ecological, evolutionary & environmental sciences

For a reference copy of the document with all sections, see nature.com/documents/nr-reporting-summary-flat.pdf

Life sciences study design

All studies must disclose on these points even when the disclosure is negative.

Sample size	Unless mentioned otherwise, experiments were repeated at least three times. MTT and AST/ALT measurements were performed in triplicates and average was taken as a single data point. Average, standard deviation and p-values are shown for each experiment and calculated by two tailed student T-test. Immunoblotting was calculated by non-parametric tests owing to the non-linearity of this method.<br>Animal studies were performed on cohorts at a size large enough to establish a statistical significane, as indicated in the methods.
Data exclusions	No data were excluded
Replication	All data was replicated in multiple cell types and for multiple sERr pharmacological combinations.
Randomization	Animals were divided into treatment cohorts prior to tumor appearance to ensure a blind randomization.
Blinding	The in vivo experiment was performed in a semi-blind fashion, in which technicians injected the mice and the analysis was done by students that are not familiar with the outcome.

Reporting for specific materials, systems and methods

We require information from authors about some types of materials, experimental systems and methods used in many studies. Here, indicate whether each material, system or method listed is relevant to your study. If you are not sure if a list item applies to your research, read the appropriate section before selecting a response.

Materials & experimental systems

n/a	Involved in the study	
☐	☒	Antibodies
☐	☒	Eukaryotic cell lines
☒	☐	Palaeontology
☐	☒	Animals and other organisms
☒	☐	Human research participants
☒	☐	Clinical data

Methods

n/a	Involved in the study	
☒	☐	ChIP-seq
☐	☒	Flow cytometry
☒	☐	MRI-based neuroimaging

Antibodies

Antibodies used	The following primary antibodies were used: mouse anti p-Tyr(PY99) (Santa Cruz Biotechnology #sc-7020, 1:500) , mouse anti-FLAG (M2) antibody (Sigma-Aldrich #F1804, 1:500), rabbit anti-KIT antibody (cell signaling #3074, 1:1000), rabbit anti p-KIT (Tyr719) antibody (cell signaling #3391, 1:1000), rabbit anti-PERK (cell signaling #5683, 1:1000), rabbit anti-IRE1 antibody (cell signaling #3294, 1:1000), rabbit anti-EGFR antibody (cell signaling #4267, 1:1000), rabbit anti p-EGFR (Tyr1068) antibody (cell signaling #2234, 1:1000), rabbit anti-ERp44 antibody (cell signaling #2886, 1:1000), rabbit anti human serum (Sigma-Aldrich # H3383, 1:50), mouse anti-MET antibody (cell signaling #3127, 1:500), rabbit anti p-MET(Tyr1234/1235) antibody (cell signaling ##3077, 1:1000), rabbit anti-PDI antibody (cell signaling #3501, 1:1000),mouse anti ATF6 antibody (Imgenex #IMG-273, 1:250), rabbit anti-α/β tubulin (cell signaling, #2148, 1:1000), anti HIF-1α (D2U3T) rabbit mAb (cell signaling #14179, 1:500), rabbit anti HSP70 (cell signaling # #4872, 1:500) , rabbit anti LC3B (cell signaling # 2775, 1:1000), MICA(2C10) (SANTA CRUZ Biotechnology #

	sc-23870, 1:250), , rabbit anti α1 antitrypsin (abcam #ab129354, 1:500), Peroxidase Wheat Germ Agglutinin (Vector laboratories #PL-1026, 1 μg/ml), Concanavalin A peroxidase conjugate (Sigma-Aldrich #L6397, 1 μg/ml), polyclonal rabbit anti p97 (1:1000) and HC70 (1:1000) were provided by Dr. Ariel Stanhill (Open University, Israel). Secondary HRP-conjugated goat anti-rabbit (1:10000) and anti-mouse (1:10000) (Jackson Immunoresearch, West Grove, PA) were used.
Validation	All antibodies were validated by the manufacturer. We have generated syngeneic KO cells that verify the manufacturer's validation.

Eukaryotic cell lines

Policy information about cell lines

Cell line source(s)	Mel526 (NIH), HepG2 (ATCC), Mel624 (NIH), HCC827 (ATCC), hTERT (Hidetoshi Tahara), and 293T (ATCC), HMC-1.1 was provided by Prof. Francesca Levi Scheffer.
Authentication	C-MET and albumin expression was used to authenticate the liver source of HepG2 and hTERT. MEL cells were used from original freezings provided by Dr. Steve Rosenberg lab to Dr. Michal Lotem at our institute. KIT expression confirmed the mast cell origin of HMC1.1. We have not performed genetic validation of the cell lines.
Mycoplasma contamination	All cells were treated with BioMyc for three weeks and validated for lack of mycoplasma prior to experiments by a PCR-based Mycoplasma test kit (Biological Industries, Israel)
Commonly misidentified lines (See ICLAC register)	No misidentified lines were involve in the study.

Animals and other organisms

Policy information about studies involving animals; ARRIVE guidelines recommended for reporting animal research

Laboratory animals	Male NOD/SCID mice at the age of 2-3 months.
Wild animals	Study did not involve wild animals
Field-collected samples	Study did not involve field-collected samples
Ethics oversight	All mouse experiments were carried out under IACUC approved protocol MD-18-15472-4 signed by the chairman of the ethic committee Prof. Tal Burstyn Cohen. HU is AAALAC approved.

Note that full information on the approval of the study protocol must also be provided in the manuscript.

Flow Cytometry

Plots

Confirm that:

- [x] The axis labels state the marker and fluorochrome used (e.g. CD4-FITC).
- [x] The axis scales are clearly visible. Include numbers along axes only for bottom left plot of group (a 'group' is an analysis of identical markers).
- [x] All plots are contour plots with outliers or pseudocolor plots.
- [x] A numerical value for number of cells or percentage (with statistics) is provided.

Methodology

Sample preparation	All samples were taken from the indicated cell-lines.
Instrument	Flow cytometry was performed by BECKMAN COULTER CytoFLEX.
Software	Data collection and analysis were performed by BECKMAN COULTER CytExpert 2.3.
Cell population abundance	Since flow cytometry analysis was performed for cell-lines, populations were pure and homogeneous.
Gating strategy	For each cell-line, live population was centered at FSC/SSC diagram of the control cells (DMSO/vehicle treated). The live population was gated and the same gate remained fixed during the whole experiment. The same number of live cells were recorded for each sample.

- [x] Tick this box to confirm that a figure exemplifying the gating strategy is provided in the Supplementary Information.
